# A pilot study to assess the healing of meniscal tears in young adult goats

**DOI:** 10.1038/s41598-021-93405-3

**Published:** 2021-07-09

**Authors:** William Fedje-Johnston, Casey P. Johnson, Ferenc Tóth, Cathy S. Carlson, Arin M. Ellingson, Melissa Albersheim, Jack Lewis, Joan Bechtold, Jutta Ellermann, Aaron Rendahl, Marc Tompkins

**Affiliations:** 1 Department of Orthopedic Surgery, University of Minnesota, Minneapolis, MN USA; 2 Department of Veterinary Clinical Sciences, University of Minnesota, St. Paul, MN USA; 3 Center for Magnetic Resonance Research, University of Minnesota, Minneapolis, MN USA; 4 Divisions of Physical Therapy and Rehabilitation Science, Department of Rehabilitation Science, University of Minnesota, Minneapolis, MN USA; 5 Department of Veterinary and Biomedical Sciences, University of Minnesota, St. Paul, MN USA; 6 Tria Orthopedic Center, Bloomington, MN USA

**Keywords:** Diseases, Health care, Medical research

## Abstract

Meniscal tears are a common orthopedic injury, yet their healing is difficult to assess post-operatively. This impedes clinical decisions as the healing status of the meniscus cannot be accurately determined non-invasively. Thus, the objectives of this study were to explore the utility of a goat model and to use quantitative magnetic resonance imaging (MRI) techniques, histology, and biomechanical testing to assess the healing status of surgically induced meniscal tears. Adiabatic T1ρ, T2, and T2* relaxation times were quantified for both operated and control menisci ex vivo. Histology was used to assign healing status, assess compositional elements, and associate healing status with compositional elements. Biomechanical testing determined the failure load of healing lesions. Adiabatic T1ρ, T2, and T2* were able to quantitatively identify different healing states. Histology showed evidence of diminished proteoglycans and increased vascularity in both healed and non-healed menisci with surgically induced tears. Biomechanical results revealed that increased healing (as assessed histologically and on MRI) was associated with greater failure load. Our findings indicate increased healing is associated with greater meniscal strength and decreased signal differences (relative to contralateral controls) on MRI. This indicates that quantitative MRI may be a viable method to assess meniscal tears post-operatively.

## Introduction

Meniscal tears are common orthopedic injuries that have important clinical consequences due to the role the meniscus plays in load transmission, shock absorption, lubrication, nutrition, and proprioception of the knee^1^. Treatment of meniscal tears has changed as subtotal meniscectomy, once commonly used for meniscal tears, has been shown to increase the risk for the development of osteoarthritis^2,3^. Methods to restore the integrity of the meniscus as much as possible through repair/suturing or limited partial meniscectomy are now seen as the best surgical options to treat meniscal tears^4^. However, post-operative evaluation of meniscal healing status is challenging as noninvasive imaging methods lack precision and accuracy. This impedes clinical decision making as the state of meniscal healing is an important determinant for when a patient can safely return to activities. If healing could be more accurately determined using non-invasive modalities, then clinical decision making would become more evidence based following meniscal repair.

Magnetic resonance imaging (MRI) is the current modality of choice to identify meniscal tears. They are most commonly diagnosed with a T2-weighted MRI sequence. The tears are identified when fluid signal within the meniscus contacts the articular surface and is consistent with the signal of the synovial fluid^5^. However, for post-operative evaluation, the standard clinical MRI sequences are nonspecific and cannot distinguish between a healing and a non-healing meniscal tear^6–9^. A potential alternative approach is quantitative mapping of MRI relaxation times, which may provide a quantitative and more sensitive means to assess changes in the healing meniscus. Quantitative T1ρ, T2, and ultrashort echo time (UTE) T2* relaxation time mapping have been previously used to assess degenerative changes to the meniscus^10–14^ and subsequent healing^15,16^. T2 and UTE-T2* mapping have also been applied to assess healing of meniscal tears^17,18^. Adiabatic T1ρ mapping^19^ has been proposed as a promising alternative to traditional continuous-wave T1ρ mapping for imaging of the meniscus due to its longer relaxation times, reduced radiofrequency transmit power, and reduced sensitivity to magic angle effects^20^. To further study how changes in relaxation times relate to the healing status of a meniscal tear, validation methods such as histology and biomechanical testing and a clinically relevant animal model are needed.

Animal models have shown promise in preclinical trials to assess healing of meniscal tears^21^. Studies have used pigs, rabbits, sheep, and goats to assess healing status of injured menisci^18,22–24^. Goats are a particularly useful model for simulating human joints due to their large size and the fact that they walk with their stifles extended, more similar to humans than some other animal models^25^. Among large animals, the meniscal anatomy of the goat has been described as the most similar to humans. The healing properties of menisci in goats have also been shown to be similar to those of humans^26^. Further, previous studies on meniscal repair and healing have been done successfully using a goat model^24,26–28^.

The purpose of this study was to investigate the potential use of a goat model to assess quantitative MRI measures of healing meniscal tears. This study had four objectives: (1) to create meniscal tears with various states of healing in a goat model; (2) to characterize and quantify the healing and morphology of goat meniscal tears using histology; (3) to investigate whether quantitative MRI techniques (adiabatic T1ρ, T2, and UTE-T2* relaxation time mapping) are sensitive in detecting healing status as determined by histological evaluation; and (4) to evaluate the potential of using failure load as a quantitative measure of healing status.

## Materials and methods

### Surgery/dissection

**Figure 1:**
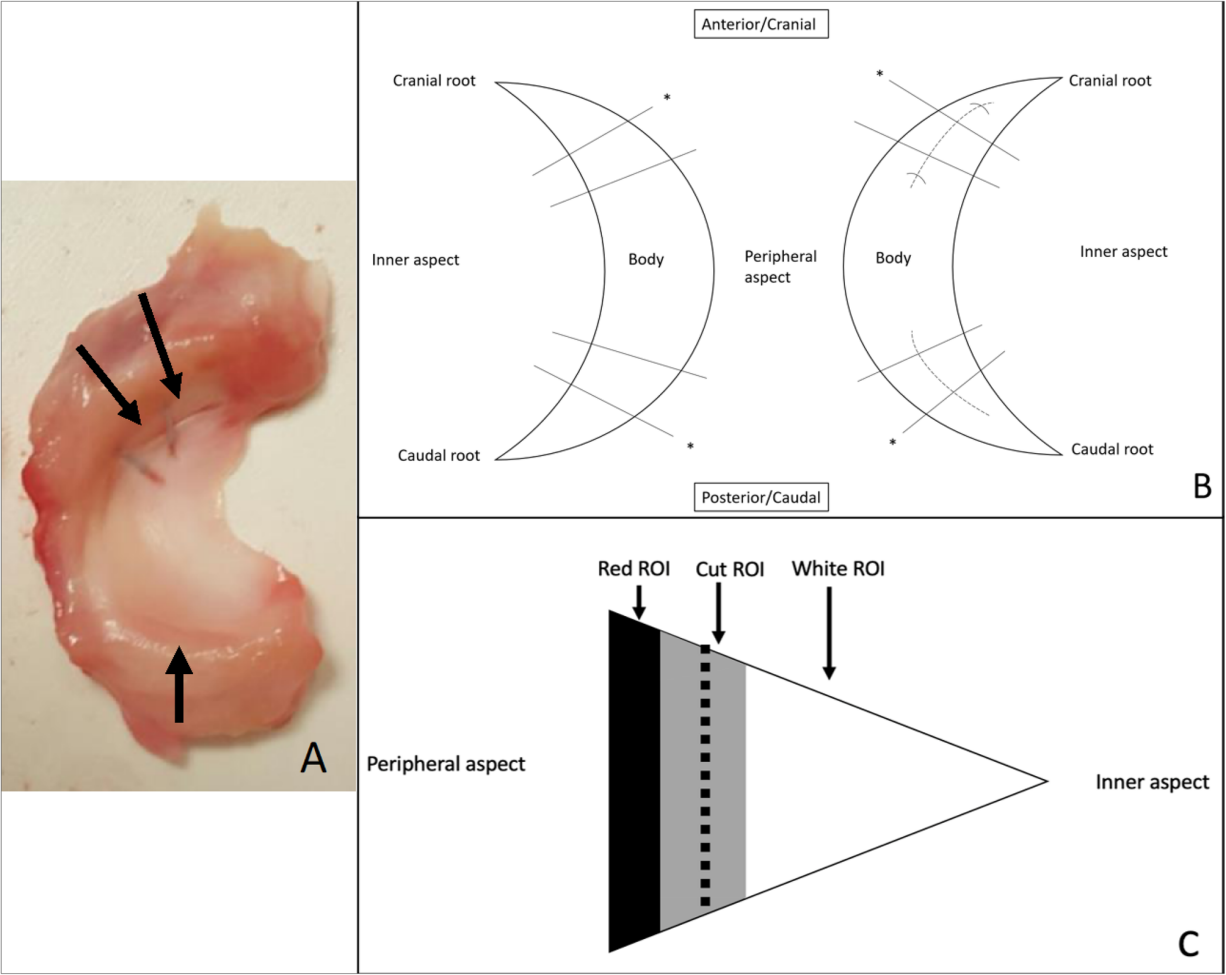
(**A**) Excised menisci from the right stifle. Operated meniscus is shown with two arrows pointing to the sutured tears on the cranial aspect (deep/inferior sutures not visible). A singular arrow shows the location of the non-repaired tear on the caudal aspect. This arrow also points in the direction of the main magnetic field (B_0_) for the MRI experiments. (**B**) Location of medial meniscal tear incisions shown with dotted lines. Cranial tears were sutured. Caudal tears were left unrepaired. Straight black lines (n = 8) represent approximate MRI imaging planes. Black lines with asterisk represent sectioning locations for histology (n = 4 per goat). Tissue between the straight black lines (3 mm in width circumferentially) was removed for biomechanical testing in the radial direction. The medial meniscus depicted on the left is the contralateral control. (**C**) Cross section of MRI imaging plane. Meniscal tear shown with dotted line. MRI regions of interest are shown as black (Red ROI), grey (Cut ROI), and white (White ROI).

Surgeries were performed on the right stifle (knee) joint of four one-year old (skeletally mature young adult) Alpine goats. The goats weighted 32.3 kg, 40.0 kg, 39.7 kg, and 45.0 kg. Procedures were reviewed and approved by the University of Minnesota Institutional Animal Care and Use Committee. All methods were performed in accordance with their guidelines and regulations. These procedures are in accordance with ARRIVE guidelines. Using a medial parapatellar approach, the patella was luxated laterally and the medial femoral condyle underwent an oblique osteotomy while maintaining attachment of the medial collateral ligament (MCL) to the condyle. Subsequent medial reflection of the condyle allowed exposure to the entire medial meniscus. Two 1 cm long, longitudinal incisions were made in the periphery of the cranial and caudal horns to simulate two longitudinal medial meniscal tears in the vascular zone (Fig. 1). Both were placed equidistant from the corresponding meniscal root and were made completely through the meniscus at 5 mm in length. Care was taken to ensure that the underlying cartilage was protected during the incision by placing a very thin metal malleable retractor under the meniscus. In a peripheral to central direction, these incisions were always placed 2 mm from the periphery such that they were within the meniscal substance, and not the capsular tissue, while also being in the peripheral 1/3 of the meniscus. The cranial (anterior) aspect was sutured using Arthrex inside out meniscus repair needles and a 2–0 Fiberwire. Two vertical mattress sutures were placed inferiorly and superiorly for a total of 4 sutures securing the tear. Sutures were placed 5 mm apart circumferentially (Fig. 1A,B). The caudal (posterior) tear was not sutured. The condylotomy was fixed with two lag screws. Goats were euthanized at 6 weeks (n = 2) and 10 weeks (n = 2) after surgery. Medial menisci were harvested from the left and right stifles and immediately frozen and stored at − 20° C for subsequent imaging, histological, and biomechanical analyses.

### Quantitative MRI

Quantitative MRI was done ex vivo at a 9.4 T field strength to allow for a high signal to noise ratio. The harvested operated (right) and control (left) medial menisci of a given animal were thawed at 4° C and mounted back-to-back in a purpose-built Teflon holder. They were mounted so the two cranial ends were next to each other and the two caudal ends were next to each other. This arrangement allowed the two menisci to be imaged simultaneously and under similar imaging conditions. Potential confounders such as variations in radiofrequency coil tuning, transmit inhomogeneity, magnetic field inhomogeneity, and magic angle effects were thus matched for the paired menisci, reducing their influences in the paired quantitative MRI data analyses. The mounted menisci were also immersed in perfluoropolyether oil (Fomblin; Specialty Fluids Company; Castaic, CA) to provide a hydrogen-free background and to eliminate magnetic susceptibility artifacts at the air-tissue interface. This enabled investigation of potential changes to the meniscal tissue at a tear site without the confounding presence of fluid signal. The menisci were analyzed ex vivo to allow for all the above controls which would not have been possible if the menisci were evaluated in vivo. The sutures were not removed to resemble the in vivo condition where sutures would remain in place during MR imaging.

The pairs of operated and control medial menisci were imaged using a preclinical 9.4 T MRI system (Agilent Technologies; Santa Clara, CA) equipped with a Varian console and millipede radiofrequency coil (Varian NMR Systems; Palo Alto, CA). Imaging included quantitative mapping of three relaxation methods: (i) adiabatic T1ρ; (ii) T2; and (iii) T2*. Adiabatic T1ρ and T2 were quantified using magnetization-prepared 2D FSE sequences at multiple imaging locations. 2D FSE imaging parameters were: FOV = 40 × 40 mm^2^ and sampling matrix = 256 × 256, or FOV = 20 × 20 mm^2^ and sampling matrix = 128 × 128; resolution = 0.16 × 0.16 mm^2^; slice thickness = 2.0 mm; TR/TE = 5000/5.4 ms; and bandwidth = 132 kHz. T2 was quantified using a double-echo preparation to generate images at echo times (TEs) of 1, 4, 8, 12, 16, and 20 ms. Adiabatic T1ρ was quantified using trains of HS1 adiabatic radiofrequency pulses with a duration of 6 ms and a maximum amplitude of 1250 Hz to generate images at spin-lock times of 0, 12, 24, 36, 48, and 60 ms. T2* was quantified using an ultrashort-echo-time (UTE) 3D radial GRE sequence with the following imaging parameters: FOV = 40 × 40 × 40 mm^3^; resolution = 0.16 × 0.16 × 0.16 mm^3^; views = 16,000; TR = 32 ms; flip angle = 5°; and bandwidth = 152 kHz; and TEs = 0.0063, 0.1, 0.2, 0.4, 0.6, 0.8, 1.0, 1.5, 2.0, 4.0, and 6.0 ms.

**Figure 2:**
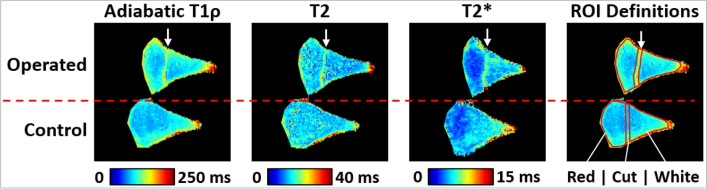
Quantitative relaxation time maps and region of interest (ROI) definitions for a representative pair of operated and control menisci at the cranial aspect of the menisci and root end of the tear site. The location of the tear is indicated by the arrow. In this example, relaxation times are increased in the vicinity of the tear site, while relaxation times in the white and red zones are similar between the operated and control menisci.

Quantitative adiabatic T1ρ, T2, and T2* relaxation time maps were generated using Matlab (version R2013b; MathWorks; Natick, Massachusetts) by fitting each set of magnetization-prepared images to a mono-exponential signal decay model on a voxel-by-voxel basis. 2D relaxation time maps that included equivalent cross-sections of the operated and control menisci were generated at four distinct locations: (i) cranial aspect of meniscus, root end of tear site; (ii) cranial aspect of meniscus, body end of tear site; (iii) caudal aspect of meniscus, root end of tear site; and (iv) caudal aspect of meniscus, body end of tear site (Figs. 1B and 2). At each of these four locations, the median adiabatic T1ρ, T2, and T2* relaxation times were calculated in three regions of interest (ROIs): (i) vicinity of tear site; (ii) white zone side of tear site; and (iii) red zone side of tear site (Figs. 1C and 2). The paired differences (Δ) between the median relaxation times of the operated and control menisci at each ROI and location were then calculated. The difference (Δ) measurements for a given ROI at the root-end and body-end of a given tear site were then averaged together to yield a single representative measurement for the tear site. This yielded adiabatic T1ρ, T2, and T2* relaxation time differences (∆relaxation time) at the cranial and caudal tear sites of each pair of operated and control menisci. The ROI measurements were then grouped by the status of the tear site (non-healed, partially healed, or healed tear) as determined by histological evaluation.

### Sectioning

Immediately after imaging, menisci were cut in the radial direction at the cranial and caudal horns of the left and right medial menisci (indicated by the asterisk in Fig. 1B). These cranial and caudal pieces (n = 4 per goat) were then fixed in 10% neutral buffered formalin prior to histological processing. Using the remaining body pieces, another cut was made at the lesion site of the cranial and caudal horns to create a 3 mm (in the circumferential direction) tissue piece (Fig. 1B) for biomechanical testing.

### Histology

#### Staining and immunostaining procedures

After fixation in 10% neutral buffered formalin, the meniscal segments intended for histology underwent routine tissue processing and were embedded in paraffin. Serial sections were taken at the cut edge of each piece, corresponding to the cranial and caudal horns (represented by lines with an asterisk in Fig. 1B) of the meniscus. 5 µm thick serial sections from all four locations (cranial and caudal cuts in right and left medial menisci) in each goat were stained with hematoxylin and eosin (H&E) to evaluate morphological features including cell nuclei, and with safranin O to evaluate proteoglycan content of the matrix. Additional serial sections were immunostained to detect type I collagen and vascular endothelium using a COL-I antibody (Sigma, St Louis, MO) and an antibody directed against factor VIII-related antigen (Dako, Carpinteria, CA), respectively. Immunostained sections were deparaffinized and rehydrated with distilled water. Type I collagen sections were treated with proteinase K, blocked with an undiluted protein block, then incubated with a mouse COL-I antibody at 1:600 for 30 min. Afterwards, sections were incubated with a secondary antibody for 30 min. The reaction product was detected with 3,3'-diaminobenzidine and sections were counterstained with Mayer’s Hematoxylin. Factor VIII-related antigen sections were treated with proteinase K, blocked with an undiluted protein block, and incubated with a factor VIII primary antibody at 1:400 for 60 min. The reaction was detected by Envision Rb + 4%NHS and sections were counterstained by Mayer’s Hematoxylin. All immunostaining procedures were done at room temperature.

#### Image analysis

The produced histological sections were imaged with a Nikon-DS-Ri2 camera attached to a Nikon Eclipse Microscope. Sections stained/immunostained for safranin O, factor VIII, and type I collagen were imaged at 1 × magnification to allow visualization of the entire section. Image analysis was done using the FIJI^29^ package in Image J. Thresholding was done for each immunostained/stained section to detect positivity. Areas of positivity were divided by the tissue area to get a percentage of immunostained/stained positivity relative to the total area of the section. This gave a proteoglycan percentage (safranin O percent positive), a vessel percentage (factor VIII percent positive), and a type I collagen percentage. Sections stained with H&E were imaged at 10 × magnification to allow visualization of cell nuclei. Two representative images were taken—one in the central third and one in the peripheral third of each meniscus section. For H&E-stained sections, cell counts of the central and peripheral thirds were added and divided by the total imaged area to obtain cell density.

#### Statistical analysis

Statistical comparisons were made using a paired t-test and a significance level set at p < 0.05. R statistical software^30^ was used to compare sutured tears with non-sutured tears, sutured tears with their contralateral control, and non-sutured tears with their contralateral control. No adjustment for multiple comparisons was done due to the exploratory nature of this study. Additionally, sections were binned according to their healing status, as defined by tissue connecting the two sides of the tear evidenced on histology. The bins were categorized as non-healed, partially healed, and healed, were averaged, and compared to their contralateral controls.

### Biomechanics

**Figure 3:**
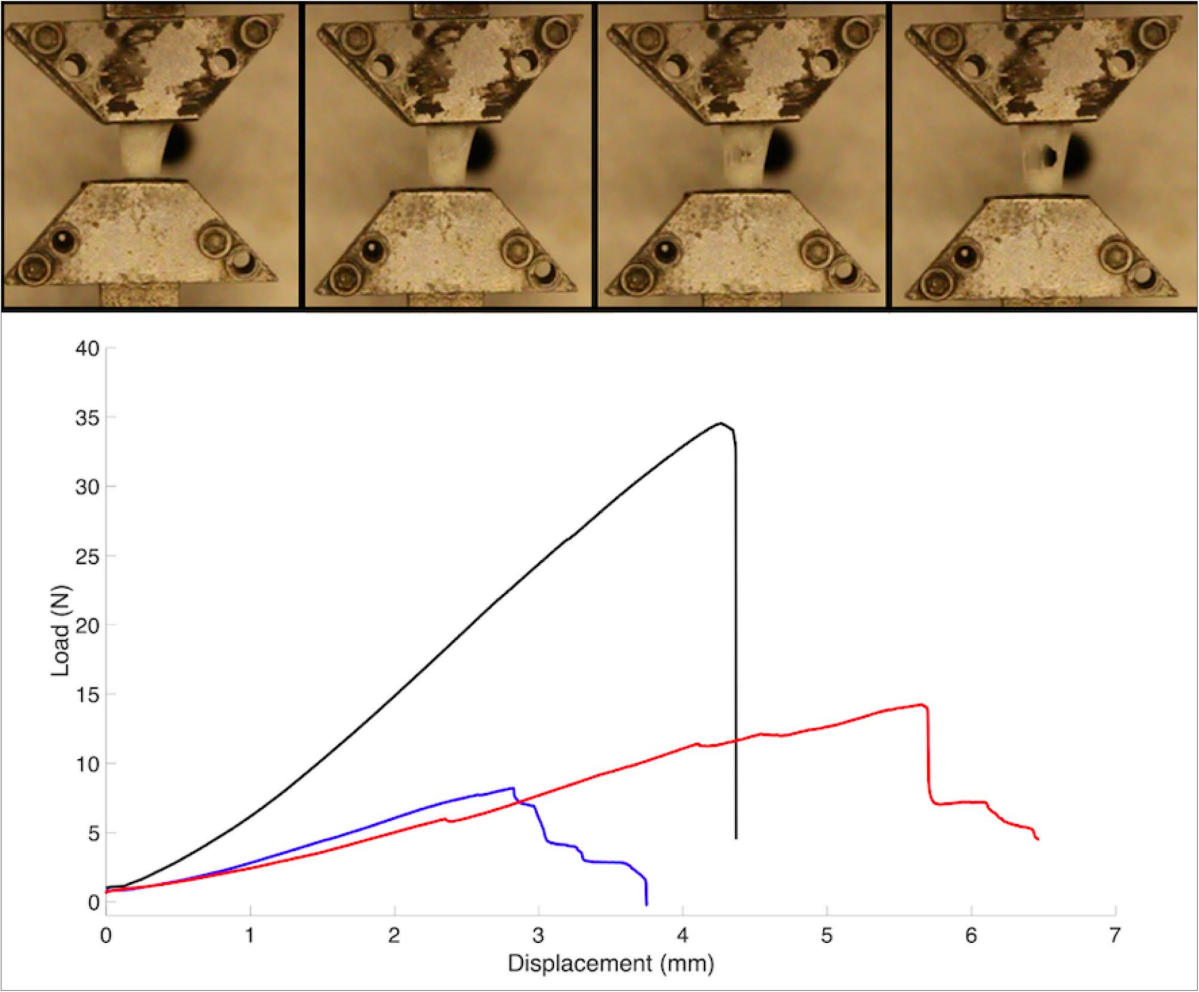
Biomechanical apparatus demonstrating the progression of tissue failure (top). Biomechanical testing of 3 different healing states (bottom): non-healed (blue), healed (red), and control (black).

Three 3 mm (measured in the circumferential direction) sections from the medial menisci of one of the goats were chosen for biomechanical testing; one section representing a non-healed repair, one representing a healed repair, and one control based on qualitative histological assessment. All three were tested biomechanically to quantify the uniaxial tensile mechanical properties in the radial direction – perpendicular to the tear site. The samples were prepared and mounted using custom grips into an Instron system (8848 Instron, Norwood, MA, USA), equipped with two dynamic load cells (Instron, Norwood, MA, USA; Range: 500 N) and loaded to failure at a rate of 0.1 mm/s (Fig. 3). Displacement was measured via the crossheads of the testing apparatus. Data were recorded at 100 Hz. Failure load and stiffness were quantified.

## Results

All condylotomies healed. There was no evidence for visible change in the articular surface of the medial compartment at the time of sacrifice.

### Histology

Based on qualitative assessment of the healing status of the meniscal tears, there was a range of healing states, as defined by tissue connecting the two sides of the tear evidenced on histology. One tear had no evidence of healing (non-healed status) and four had partial healing (partially healed status). Three of the tears had healed so well that the area of the tear was no longer visible histologically (healed status). In three of the four goats, healing of the non-sutured tears was more advanced than healing of the sutured tears. At 6 weeks, both sutured lesions were partially healed. The two unrepaired lesions were classified as partially healed and healed. At 10 weeks, the sutured lesions were non-healed and partially healed. The unrepaired lesions were both healed. In partially and fully healed lesions, the healing and vascular tissue was noted to progress from the synovium over the surface of the meniscus to the tear site, and not penetrate through the meniscal tissue itself.

#### Vascular percent

**Figure 4:**
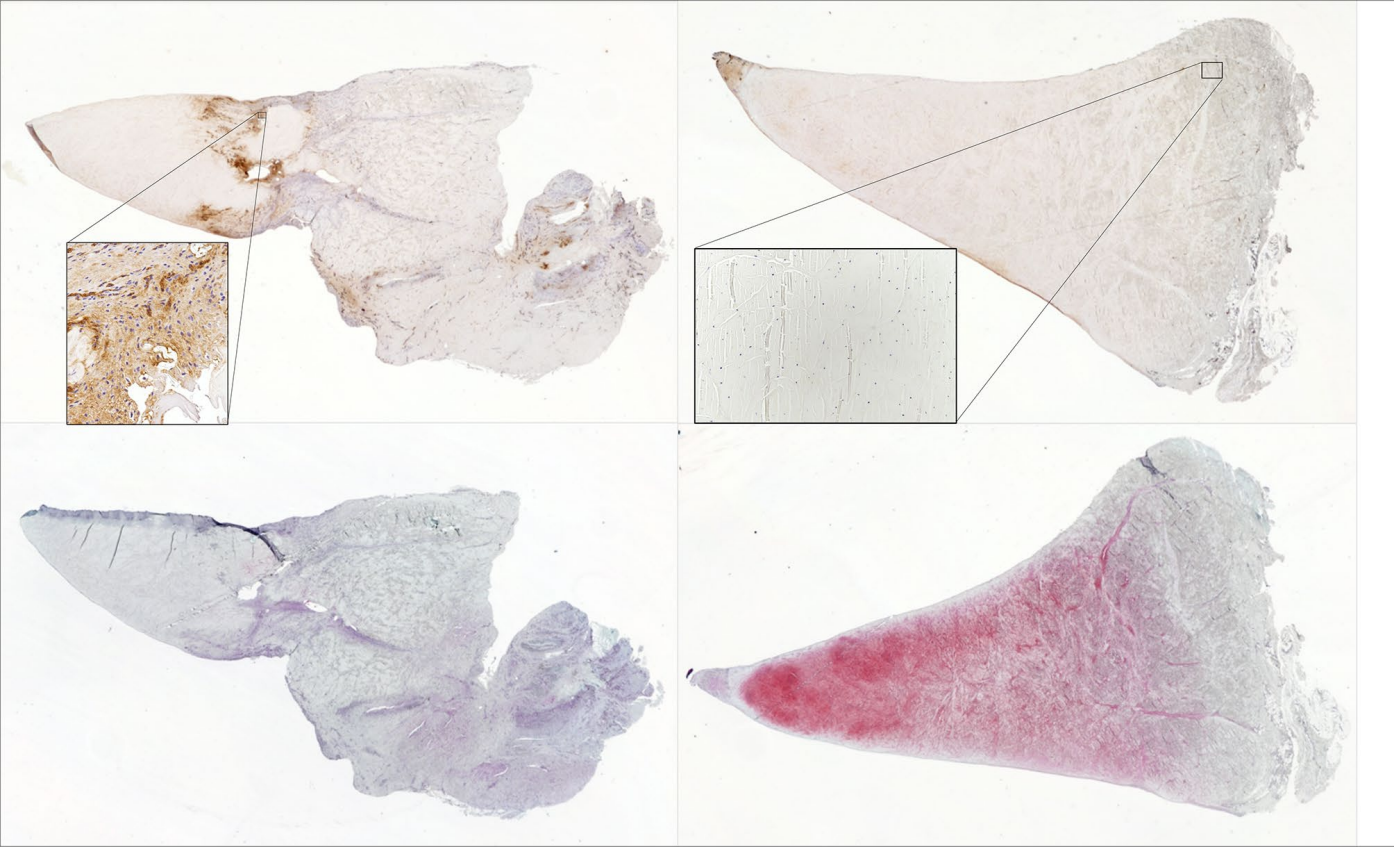
Comparison of sutured section six weeks post-operative with contralateral control. Serial sections were immunostained with factor VIII-related antigen (top images); the reaction product is brown. Insets demonstrate vessels with endothelial cells positive for factor VIII-related antigen (top left) and area containing no vessels (top right). Complete tissue sections are 1 × objective and insets are 20 × objective. Additional sections were stained with safranin-O (bottom images); red staining indicate the presence of proteoglycans.

Factor VIII-related antigen positivity was higher around the tear sites when compared to their contralateral controls. Percent positivity was significantly (p = 0.04) greater in the sutured location (1.02%) than its contralateral control (0.0235%) (Fig. 4, Table 1). Percent positivity for factor VIII-related antigen was also significantly (p = 0.02) greater in the non-sutured tear site (0.700%) than in its contralateral control (0.0216%). The increased percentage of vessels was due to vascular proliferation surrounding the tear site (Fig. 4). No significant difference in means was found between sutured and non-sutured tear sites.

**Table 1:** Mean values of morphological features for sutured, non-sutured, and their respective contralateral control locations.

Location	n	Mean vascular percent^†^	Mean proteoglycan percent^†^	Mean cell density (cells/mm^2^)^†^	Mean collagen percent^†^
Cranial (sutured)	4	1.02 ± 0.608	8.31 ± 13.2	1230 ± 565	10.2 ± 12.4
Cranial contralateral control	4	0.0235 ± 0.0208	55.7 ± 10.5	602 ± 298	6.38 ± 4.38
Caudal (non-sutured)	4	0.700 ± 0.302	5.79 ± 5.99	993 ± 595	9.63 ± 18.5
Caudal contralateral control	4	0.0216 ± 0.0252	34.3 ± 15.6	488 ± 94	12.4 ± 11.9

Data represents both 6- and 10-week time periods as significant differences between these time periods were not observed. Values are reported with their Standard Deviation.

^†^Values represent percentage of stain/immunostain positivity relative to the area of the section (or total area evaluated).

**Figure 5:**
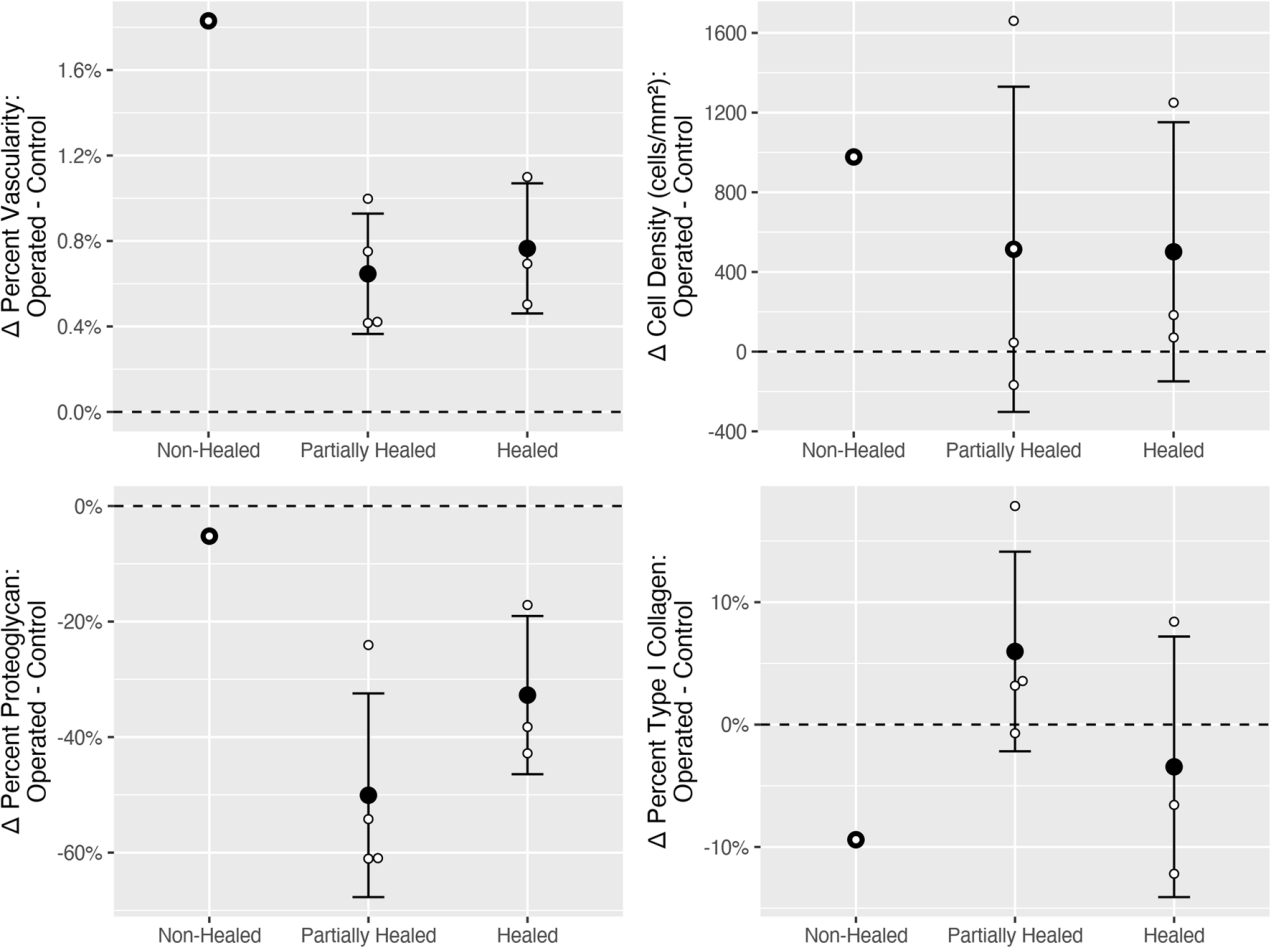
Average difference in morphological parameters between induced lesions (sutured/non-sutured) and contralateral control meniscal sections. Vascularity, cell density, proteoglycan content (safranin-O), and type I collagen were binned according to the healing status (non-healed, partially healed, and healed tears). The average difference between non-healed (n = 1), partially healed (n = 4), and healed (n = 3) tears and their respective contralateral control are shown with standard deviation bars. Cell density was the difference in cells per mm^2^. All other parameters are differences in percent stain/immunostain positivity. Non-healed tear does not have error bars as there was only one tear with this status. Note the dotted black line indicating no difference between induced lesions and their contralateral control.

After binning according to healing status (defined as the completeness of tissue connection at the tear site in histology), non-healed status was the most different from its contralateral control (Fig. 5, top left) regarding vascular percent. The other two states, partially healed and healed, were more similar to their contralateral control than the non-healed status (Fig. 5, top left).

#### Cellular density

No significant differences in mean cellular density were observed between sutured and non-sutured tear locations with a 95% confidence interval between − 624 and 1099 cells/mm^2^. Further, no significant differences in means were observed between sutured tears and their contralateral control locations (95% confidence interval between − 721 and 1979 cells/mm^2^) nor between non-sutured tears and their contralateral control locations (95% confidence interval between − 340 and 1350 cells/mm^2^).

When comparing cellular density for the various healing states with the contralateral controls, non-healed tears had the greatest difference (Fig. 5, top right). Partially healed and healed tears had similar differences from their contralateral controls (Fig. 5, top right).

#### Proteoglycan percent

Menisci with induced tears had a marked decrease in proteoglycan positivity relative to their contralateral controls (Fig. 4). The sutured tear site had significantly (p = 0.03) less safranin-O positivity than its contralateral control (mean of 8.31% versus 55.7%, respectively) (Fig. 4, Table 1). The non-sutured tear site had significantly (p = 0.01) less safranin-O positivity than the contralateral control (mean of 5.79% versus 34.3%, respectively).

When comparing the three types of healing status with their contralateral controls, differences in proteoglycan content decreased in the following order: partial > healed > non-healed tear (Fig. 5, bottom left).

#### Collagen percent

Mean differences in percent type I collagen immunopositivity between sutured tear site (10.2%) and non-sutured tear site (9.63%) were not significant (p = 0.88). Mean differences between sutured tear site (10.2%) and its contralateral control site (6.38%) were not significant (p = 0.54). Lastly, mean differences between non-sutured tear site (9.63%) and its contralateral control site (12.4%) were not significant (p = 0.57).

When assessed according to healing status, type I collagen immunopositivity was most different between tear site and contralateral control and decreased in the following order: non-healed > partially healed > healed tear (Fig. 5, bottom right).

### Quantitative MRI

**Figure 6:**
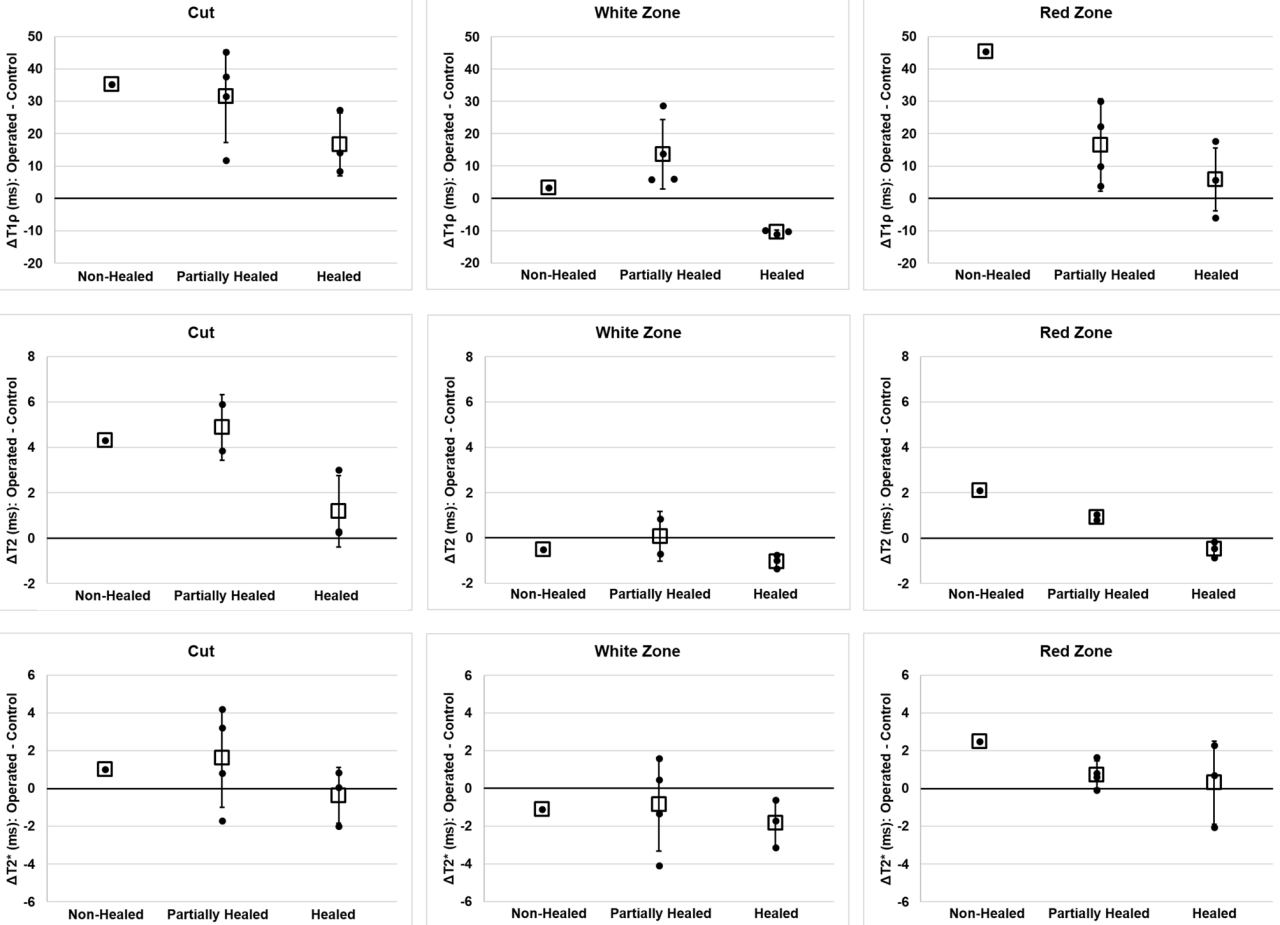
Relaxation time differences (Δ) between the operated and control menisci. The relaxation time differences were calculated in three ROIs: near the tear site (first column); the white zone side of the tear site (second column); and the red zone side of the tear site (third column). Three relaxation times were evaluated: adiabatic T1ρ (first row); T2 (second row); and T2* (third row). Individual data points are shown as black dots, their mean is plotted as a square, and the error bars show the standard deviation of the mean. Since there was only one non-healed tear, there are no error bars for this point.

Using the classifications of non-healed, partially healed, and healed tears as assessed histologically, the mean differences between operated and contralateral control relaxation times (∆relaxation time) were calculated for adiabatic T1ρ, T2, and T2* at all three ROIs (Fig. 1C). The ∆relaxation times for each ROI are plotted in Fig. 6, and quantitative results are tabulated in Table 2.

**Table 2:** Quantification of the three relaxation times in each of the three regions of interest and for each of the three healing states.

MRI method	Healing state	N	Control (ms)	Operated (ms)	Paired Difference (ms)	Percent increase
**Cut site**						
Adiabatic T1ρ	Non-healed	1	79	114	35	45%
	Partially healed	4	70 ± 21	102 ± 34	32 ± 14	43 ± 12%
	Healed	3	76 ± 10	93 ± 20	17 ± 10	21 ± 9%
T2	Non-healed	1	11.7	16.0	4.3	37%
	Partially healed	2	14.4 ± 1.8	19.3 ± 0.4	4.9 ± 1.4	35 ± 15%
	Healed	3	13.2 ± 1.8	14.4 ± 3.3	1.2 ± 1.6	8 ± 10%
T2*	Non-healed	1	5.3	6.3	1.0	19%
	Partially healed	4	4.7 ± 0.3	6.3 ± 2.4	1.6 ± 2.6	37 ± 57%
	Healed	3	5.2 ± 0.1	4.8 ± 1.4	− 0.4 ± 1.5	− 7 ± 28%
**White zone**						
Adiabatic T1ρ	Non-healed	1	90	93	3	4%
	Partially healed	4	77 ± 20	91 ± 26	14 ± 11	17 ± 12%
	Healed	3	89 ± 12	79 ± 13	− 10 ± 1	− 12 ± 2%
T2	Non-healed	1	12.4	11.9	− 0.5	− 4%
	Partially healed	2	13.8 ± 1.0	13.9 ± 2.1	0.1 ± 1.1	0 ± 8%
	Healed	3	13.5 ± 1.8	12.5 ± 2.0	− 1.0 ± 0.3	− 8 ± 3%
T2*	Non-healed	1	7.4	6.3	− 1.1	− 15%
	Partially healed	4	7.0 ± 2.0	6.2 ± 3.0	− 0.9 ± 2.5	− 10 ± 38%
	Healed	3	6.3 ± 0.6	4.5 ± 0.7	− 1.8 ± 1.3	− 28 ± 17%
**Red zone**						
Adiabatic T1ρ	Non-healed	1	63	109	45	72%
	Partially healed	4	68 ± 24	85 ± 34	17 ± 12	22 ± 12%
	Healed	3	72 ± 15	78 ± 8	6 ± 12	10 ± 17%
T2	Non-healed	1	12.1	14.2	2.1	17%
	Partially healed	2	14.1 ± 1.0	15.0 ± 1.2	0.9 ± 0.2	7 ± 1%
	Healed	3	14.3 ± 3.5	13.8 ± 3.1	− 0.5 ± 0.4	− 3 ± 2%
T2*	Non-healed	1	3.7	6.2	2.5	68%
	Partially healed	4	4.6 ± 0.4	5.3 ± 0.9	0.7 ± 0.7	16 ± 16%
	Healed	3	4.4 ± 0.4	4.7 ± 1.8	0.3 ± 2.2	10 ± 51%

Values are shown as mean ± standard deviation.

Adiabatic T1ρ relaxation times were considerably increased in the operated vs. control menisci at the cut site ROI and in the red zone ROI for both non-healed and partially healed tears. The **∆**relaxation times were notably lower in the healed tears, which suggests that adiabatic T1ρ relaxation times in the cut and red zones may be normalizing as healing progresses. There was no clear relationship between **∆**relaxation times and healing status in the white zone ROI.

T2 relaxation times followed a similar trend as the adiabatic T1ρ findings. The cut and red zone ROIs had increased T2 relaxation times in the operated vs. control menisci for the non-healed and partially healed tears, while little difference was detected in the healed tears. No notable ∆relaxation times were observed among the healing states in the white zone ROI.

T2* relaxation times in the operated vs. control menisci were also greater in the non-healed and partially healed tears than the healed tears in the cut site ROI. The red zone ROI also had a decrease in ∆relaxation time with increased healing status. No clear differences in ∆relaxation times were observed in the white zone ROI.

### Biomechanics

The control section reached failure at 34.5 N and had a stiffness of 8 N/mm. The healed section reached failure at 14.2 N with a stiffness of 2.7 N/mm. The non-healed section reached failure at 8.2 N with a stiffness of 2.9 N/mm (Fig. 3).

## Discussion

The results of this exploratory study support the hypotheses that quantitative MRI methods can be used to determine the healing status of meniscal tears and that the MRI results are supported by histological and biomechanical data. Histology allowed a classification of tears as non-healed, partially healed, or healed. Based on these healing states, we saw a decrease in **∆**relaxation time with increased healing in adiabatic T1ρ, T2, and T2* in both the cut and red zone ROIs (Fig. 6); there were no discernable trends in the white zone. Biomechanical testing paralleled the MRI data as we observed greater failure load concomitant with better healing status.

The utility of this goat model for longitudinal meniscal tears in medial meniscus injury was encouraging in this study. The menisci exhibited variable healing states, which is similar to human meniscal tears. There were, however, findings that were different in goats than we would expect in humans. First, there were no discernable differences between 6- and 10-week time points, suggesting that meniscal healing in goats occurs much more rapidly than in humans. Second, there were differences in the healing potential in different meniscal locations. The cranial horn did not heal as well as the caudal horn even though it was sutured, and the caudal horn was not. This has been observed previously^28^, and may be related to the loading of the goat stifle joint, which is different than the human knee joint. While similar, there are established differences between goat and human meniscal and knee anatomy^31^. Both the width and length of the goat meniscus are smaller than humans and there are differences in surrounding ligaments and passive range of motion^31,32^. Despite these potential differences between goats and humans, the goat model is still promising as it bears a close resemblance to human knee anatomy and has similar vascular properties^25,26,32^. Analysis of a wider range of time points and anatomical locations are needed to fully realize the potential of goats as animal models for meniscal healing.

Biomechanical and histological findings offered insights into the healing process of menisci that are similar to results from previous studies and suggest the goat model can be used to evaluate meniscal healing^18,22,23^. Biomechanically, greater failure loads were observed with greater healing status which is consistent with other studies^18,23^. The histological features of the menisci changed with the introduction of the meniscal tears. The number of vascular profiles, assessed both with routine H&E sections and using immunohistochemical detection of factor VIII-related antigen to identify endothelial cells, markedly increased around the lesion site when compared to the contralateral control site. This increase in vascularity surrounding the lesion site has also been observed in previous histological studies in dogs and rabbits^22,33,34^. In these studies, the vessels were interpreted to arise from the synovium, which is consistent with our findings. In additional histological findings, there was a marked decrease in the proteoglycan content of the operated menisci as assessed by safranin O staining. This result is consistent with a previous study done in rabbits where transection of both menisci and the caudal cruciate ligament resulted in decreased safranin O staining^35^.

Our findings support the hypothesis that quantitative mapping of adiabatic T1ρ, T2, and/or T2* relaxation times may be useful to assess meniscal healing status. All three relaxation times in the operated menisci were closer to the control menisci values in the healed vs. partially healed cut sites. This suggests that the relaxation times are normalizing as healing progresses, which would potentially provide a quantitative means to assess the degree of meniscal healing. The relaxation times were also increased in the operated vs. control menisci in the non-healed cut sites. This suggests, given the absence of synovial fluid, that there are changes to the tissue and/or extracellular matrix near the cut site in response to injury that remain if the lesion is not repaired. Interestingly, the relaxation times in the red zone ROI also follow a similar (albeit relatively reduced) response to healing, while there were no notable changes in the white zone ROI. The increase in relaxation times at the cut site may be driven in part by the vascular response observed in the histology results, which was shown to be more prominent in non-healed and partially healed tear sites. An increase in vasculature and/or fibrovascular tissue may contribute to longer relaxation times (e.g., due to longer relaxation times of the blood compartment). The changes may also represent degenerative changes at the cut site and red zone. Degeneration of the meniscus, which includes fraying, disorganization of collagen fibers, and increased water content, causes an increase in T1ρ, T2, and T2* relaxation times in humans^10,11,36^.

Our findings of increased relaxation times following a meniscal tear and normalization with healing progression are consistent with prior reports. In an in vivo ovine model study at 3 T MRI, Koff et al*.* found that T2* relaxation times were increased in operated menisci 4 and 8 months following injury compared to contralateral-control menisci, but there was limited healing in this time interval^18^ . In an in vivo human study at 7 T MRI, Stelzeneder et al*.* found that T2* relaxation times were increased 6 months post-operatively in torn menisci repaired with suturing compared to control menisci, but T2* values were then reduced at 12 months post-operatively^17^. Yamasaki et al. found, in an in vivo study at 3 T MRI, that T2 relaxation times were reduced in healed vs. non-healed meniscal tears 6 to 36 months after its repair compared to pre-operative values^37^. Our findings warrant further investigation to confirm the relationship between MRI relaxation times and healing of meniscal tears. Additionally, determining precisely what structural, biochemical, and/or morphological changes these relaxation times are sensitive to is warranted.

The quantitative MRI findings are potentially clinically translatable. The investigated relaxation time mapping methods have been previously applied for in vivo imaging of menisci and/or articular cartilage of the knee at both 3 T and 7T^9,12,14,17,20,37–40^. One potential issue is obtaining sufficient spatial resolution and signal-to-noise ratio to quantify relaxation times in the vicinity of the tear site. However, given that (i) tears are discernable on T2-weighted images, (ii) changes to the red zone may reflect changes to the tear site, and (iii) 7 T is now a clinical modality for the knee, we anticipate the requisite resolution will be achievable. Another factor to consider is the potential need to apply fluid suppression to remove any confounding influence of synovial fluid at the tear site (which has long relaxation times). Granted, the presence of synovial fluid at the tear site may indicate that the tear is not sufficiently healed and thus serve to further distinguish healing states. Refining the relaxation time mapping techniques and validating them with additional animal model work, followed by clinical studies in humans, would provide the needed information to allow physicians to make informed decisions regarding (post-operative) management of meniscal tears using MRI.

There are several limitations to our exploratory study, the primary one being a small sample size. For biomechanical testing, we were not able to test all specimens, resulting in a further decrease in sample size. Because the MRI work was done ex vivo, translation to a live imaging model is necessary to support clinical relevance. We were also unable to obtain a wide spectrum of healing based on healing time. The time points that we chose (6 weeks and 10 weeks) did not provide enough of a difference to see chronologic changes in healing. Lastly, creation of the meniscal tears required an invasive osteotomy procedure, but this could be changed to an arthroscopic model in the future.

In summary, this study expands upon previous studies, offers new insights, and demonstrates potential future directions for meniscal healing studies using a goat model. In addition, this study suggests a potential for quantitative MRI relaxation time mapping to provide useful information in determining meniscal healing status.

## Data Availability

The datasets generated during and/or analyzed during the current study are available from the corresponding author on reasonable request.
